# Clinicopathological and ultrasound features as risk stratification predictors of clinical and pathological nodal status in papillary thyroid carcinoma: a study of 748 patients

**DOI:** 10.1186/s12885-022-09474-8

**Published:** 2022-04-01

**Authors:** Cui Zhang, Baojun Li, Lei Zhang, Fengjiao Chen, Yanhua Zhang, Wen Cheng

**Affiliations:** 1 Department of Medical Ultrasound, Harbin Medical University Cancer Hospital, Harbin, 150081 Heilongjiang China; 2 Department of Head and Neck Surgery, Harbin Medical University Cancer Hospital, Harbin, 150081 Heilongjiang China; 3 Department of Interventional Ultrasound, Harbin Medical University Cancer Hospital, Harbin, 150081 Heilongjiang China

**Keywords:** Papillary thyroid carcinoma, Intermediate risk, Clinicopathology, Ultrasonography, Risk stratification

## Abstract

**Background:**

Papillary thyroid carcinoma (PTC) is the most common histological type of thyroid malignancy that tends to metastasize to cervical lymph nodes. In the present study, we aimed to investigate which clinicopathologic and ultrasound features of PTC are associated with clinical lymph node metastasis (LNM) and numbers of pathological LNM.

**Methods:**

From January 2016 to December 2018, we identified a cohort of patients with PTC who underwent cervical ultrasonography and were diagnosed through operation and pathology. Clinical N1(cN1) and > 5 pathologic N1(pN1) were considered in the postoperative stratification to have an intermediate risk according to the 2015 ATA guidelines. Clinicopathological and ultrasound features in PTC patients were performed in accordance with the independent risk factors of cN1 and > 5pN1 respectively by using the univariate and multivariate analyses.

**Results:**

We collected 748 PTC patients in the final inclusion criteria. There were 688 cN0 cases and 60 cN1 cases. From the analyses, primary tumor size > 2 cm, capsule contact, extrathyroidal extensions (ETE) and central LNM remained independent risk factors for cN1 in PTC patients. In the 748 PTC patients, 707 cases had ≤ 5 pN1, and 41 cases had > 5 pN1. Multifocality, primary tumor size > 2 cm, capsule contact and ETE are significant independent risk factors for > 5 pN1.

**Conclusions:**

We concluded that multifocality, primary tumor size > 2 cm, capsule contact, ETE and central LNM were independent risk factors for the intermediate risk stratification in patients with PTC. Ultrasonography is a good technique for the preoperative lymph node staging of PTC and is helpful for detecting LNM.

## Background

Papillary thyroid carcinoma (PTC) is the most common histological type of thyroid malignancy and tends to metastasize to the cervical lymph nodes (LNs). Fortunately, PTC rarely becomes life threatening, and patients mostly have excellent clinical outcomes after surgery. Lymph node metastasis (LNM) is common in thyroid carcinoma. A number of previous studies have reported that the incidence of central or lateral lymph node metastasis in PTC is approximately 23.0–51.7%. Previous studies demonstrated that the size and number of lymph node metastases based on preoperative imaging studies and postoperative pathology significantly reflect patient prognosis [1–3].

The most recent tumor-node-metastasis staging system 8th edition of the American Joint Commission on Cancer (AJCC 8th) now stages differentiated thyroid cancer (DTC) based only on the presence or absence of LN involvement without specifying the location or the number of LNs [4]. In the 2015 American Thyroid Association (ATA) guidelines [5], the risk stratification system has three tiers and uses clinicopathologic features available at the time of initial treatment to classify DTC patients as having either a low, intermediate or high risk of either recurrence or persistent disease. The low-risk and intermediate-risk PTC subgroups are common clinically. The recurrence risk intermediate-risk PTC is approximately 20%. The intermediate-risk PTC subgroup has more aggressive disease and experiences more recurrence or distant metastasis from LNM than the low-risk PTC subgroup. The high-risk PTC subgroup undoubtedly has the most aggressive disease. However, with the popularization of physical examinations, high-risk PTC is rarely seen clinically.

Recently, clinical N1 (cN1) and > 5 pathologic N1 (pN1) were considered in the postoperative stratification to have an intermediate recurrence risk according to the 2015 ATA guidelines. And the risk factors cN1 and > 5 pN1 were not included in the original 2009 initial risk stratification system. Once identified as having an intermediate-risk, postoperative treatment, such as radioiodine administration and follow-up, may vary.

To differentiate patients with a low risk from patients with an intermediate risk, if possible, at a presurgical stage, it is essential to appropriately evaluate the clinicopathological and ultrasound features of PTC in an attempt to better predict patient prognosis and offer more appropriate treatment. Additionally, previous studies demonstrated that clinical and pathologic node metastasis is a strong prognostic factor [6]. Regarding LNM in PTC, there have been no previous studies on the association of the risk factors of cN1 and > 5 pN1 with LNM. In the current study, we hypothesized that clinical LNM and numbers of pathological LNM were potentially associated with intermediate risk stratification in patients with PTC. To better characterize patients based on lymph node involvement, the present study aimed to investigate which clinicopathologic and ultrasound features of PTC are associated with clinical LNM and the number of LNM.

## Methods

### Study cohort

A total of 1136 PTC patients between January 2016 and December 2018 at Harbin Medical University Cancer Hospital were enrolled. Initially, we reviewed the medical records of 1136 PTC patients.

Patients were enrolled if they met all of the following inclusion criteria: (1) the patients underwent initial total thyroidectomy or subtotal thyroidectomy or lobectomy; (2) the patients underwent ipsilateral or bilateral central lymph node dissection (LND) (level VI) with or without lateral LND, and at least one lymph node was removed from the central compartment (level VI); and (3) the patients had no preoperative evidence of distant metastasis.

The exclusion criteria primarily consisted of other pathologic types of thyroid cancer, pT4a gross ETE, pN1 with any metastatic LNs ≥ 3 cm along the largest diameter (high-risk structural disease recurrence according to the ATA), pN1 with extranodal extension (ENE) and > 3 LNs involved (high-risk structural disease recurrence according to the ATA), a history of thyroid or neck surgery, another primary tumor, a history of irradiation involving the neck region, and cases missing important data.

Finally, we included 748 PTC patients (median age, 48 years; range, 19–74 years; 640 women and 108 men). The preoperative LNM status was evaluated with high‐resolution neck ultrasound(US).

The Institutional Review Board of Harbin Medical University Cancer Hospital approved this study. The need for informed consent was waived because of the retrospective nature of the cohort study.

### Clinicopathological and US features

All patients were initially assessed with an Aixplorer US system (Super Sonic Imagine, Aix-en-Provence, France) equipped with a linear-array probe of 15–44 MHz by two radiologists with 7 years of experience in thyroid US. We obtained data including capsule contact, Chinese thyroid imaging reporting and data system (C-TIRADS) and shear wave elastography (SWE) imaging of the suspicious lesion.

The C-TIRADS features of the thyroid lesions were regarded as suspicious US features for malignancy: vertical orientation, ill-defined or irregular margins (including extrathyroidal extension), microcalcifications, solid, and markedly hypoechoic [7].

### Shear Wave Elastography (SWE) Imaging

After conventional US, elastography information was obtained according to the quantitative elasticity index on SWE. SWE values were routinely obtained and recorded, including the maximum (E_max_) and mean (E_mean_) elasticity values (kPa) and the elastic ratio (ER, the mean stiffness for the lesion to the normal parenchyma).

According to the 2015 ATA guidelines, cN1 and > 5 pN1 were considered to have an intermediate risk stratification for structural disease recurrence in patients with PTC.cN1 was defined as the presence of suspicious neck LNs identified by preoperative physical examination, preoperative imaging (US and/or CT), and/or intraoperative inspection. > 5 pN1 was defined as more than 5 metastatic lymph nodes in postoperative pathology.

All data were classified depending on whether they were cN1 (subdivided into cN0 and cN1) and whether they were > 5 pN1 (subdivided into > 5 pN1 and ≤ 5 pN1). Through the retrospective review of clinical data and pathologic reports, clinicopathological and US features were recorded. Clinicopathological and US features in PTC patients were conducted to find the risk factors of cN1 and > 5pN1 respectively.

The clinicopathological and US factors of cN1 in PTC included age, sex, Hashimoto's thyroiditis (HT), nodular goiters, multifocality, bilaterality, primary tumor size, extrathyroidal extensions (ETE), capsule contact, SWE, central LNM, 5pN1.The clinicopathological and US factors of 5pN1 in PTC included age, sex, HT, nodular goiters, multifocality, bilaterality, primary tumor size, ETE, capsule contact, SWE, C-TIRADS grade, central LNM.

### Thyroid surgery

All patients underwent total or subtotal thyroidectomy or lobectomy with bilateral or ipsilateral central neck dissection with or without lateral neck dissection. Patients underwent subtotal thyroidectomy or lobectomy with ipsilateral central neck dissection. Central neck dissection includes dissection of the pretracheal, prelaryngeal, and ipsilateral paratracheal LNs. Lateral neck dissection was performed when at least one lateral lymph node suspicious for metastasis was detected on preoperative US or confirmed through US findings, fine needle aspiration (FNA) cytology, or surgical exploration. The histopathologic results were reviewed for pathologic TNM staging and the status of neck lymph node metastasis. The number of pN1 lesions was calculated including both lateral compartment lymph nodes (level II, III, IV, And V) and ipsilateral or bilateral central compartment lymph nodes (level VI).

### Statistical analysis

SPSS 20.0 software was used for statistical analysis, and bilateral test results are given (test level α = 0.05). *p* < 0.05 was considered statistically significant. Statistical analysis of the collected data was performed, and univariate analysis was conducted for different groups to identify risk factors related to lymph node metastasis. The T' test was used to analyze the homogeneity and heterogeneity of variance. For count data, N (%) values and chi-square tests were used for analysis. Variables with statistically significant distribution differences among different groups in univariate analysis were included in the multivariate logistic model to explore independent influencing factors related to lymph node metastasis.

## Results

A detailed presentation of the clinicopathological characteristics of patients at initial treatment for PTC can be accessed in Table 1.

**Table 1:** Clinicopathological features of patients at initial treatment of PTC

	**Number of patients**
Gender (M:F)	1:5.9
Female	640 (85.6%)
Male	108 (14.4%)
Age(years)	48.1 ± 14.2
≤ 40	246 (32.9%)
> 40 and ≤ 55	423 (56.6%)
> 55	79 (10.5%)
Tumor size, cm	0.93 ± 0.76
Thyroidectomy	
Total thyroidectomy	538 (71.9%)
Subtotal thyroidectomy or lobectomy	210(28.1%)
Lymph node dissection	
LND + CND	417 (55.7%)
CND only	331 (44.3%)
pT stage^a^	
T1	540 (72.2%)
T2	170 (22.7%)
T3	38 (5.0%)
pN stage^a^	
N0	489 (65.4%)
N1a	217 (29.0%)
N1b	42 (5.6%)

*LND* Lateral neck dissection, *CND* Central neck dissection

^*a*^ pT stage, pN stage as AJCC 8th edition Tumor-Node-Metastasis Staging System

### Demographics and clinicopathological and ultrasound features

Our cohort comprised 640 (85.56%) females and 108 (14.44%) males among the 748 patients, and the mean age of the patients was 48.1 ± 14.2 years. Seventy-nine patients were > 55 years old (10.56%). The median tumor size was 0.93 ± 0.76 cm, and 540 patients (72.19%) were papillary thyroid microcarcinoma (PTMC). 531 patients (70.99%) were pN0.HT was not frequently detected (*n* = 81, 10.82%) in cN1 PTC (*n* = 5, 0.7%) and in pN1 PTC (*n* = 4, 0.5%). Nodular goiters were found in 351 patients (46.93%). The majority of tumors were unilateral (*n* = 462, 61.76%), unifocal (*n* = 607, 81.15%) and had capsule contact (*n* = 473, 63.24%). Histological ETE was present in 243 (32.49%) patients, and 67 (27.57%) had aggressive extension to the perithyroidal soft tissue. The C-TIRADS was established based on the counting method by dividing thyroid nodules into the following categories: Category 4a (69,9.22%), Category 4b (153,20.45%), Category 4c (466,62.30%), and Category 5 (60,8.02%) based on ultrasound evaluation.

### Association between cN1 and clinicopathological features

Among the 748 included PTC patients, there were 688 cN0 cases and 60 cN1 cases. Comparisons of clinicopathological and US characteristics between the 2 groups of patients with PTC are shown in Table 2. We found a significant association between cN1 and the primary tumor size, capsule contact, ETE, Emax, ER and distribution of central LNM (*p* < 0.05). Multivariate logistic regression analysis demonstrated that primary tumor size > 2 cm (OR = 5.05 (95% CI 1.88–13.55); *p* = 0.01), capsule contact (OR = 5.77 (95% CI 1.27–26.30); *p* = 0.02), ETE (OR = 4.20 (95% CI 1.78–8.98); *p* = 0.01), central LNM (OR = 5.18 (95% CI 2.22–12.06); *p* < 0.001) and number of pN1 (OR = 9.52 (95% CI 4.04–22.43); *p* < 0.001) remained independent risk factors for cN1 in PTC patients (Table 3).

**Table 2:** Comparisons of clinicopathological features between cN0 and cN1 of PTC patients

Variable	Total (*n* = 748)	cN0 (*n* = 688)	cN1 (*n* = 60)	χ2	*p*
Age (years)				3.31	0.19
≤ 40	246	220 (32.0%)	26 (43.3%)		
> 40 and ≤ 55	423	395 (57.4%)	28 (46.7%)		
> 55	79	73 (10.6%)	6 (10.0%)		
Gender				1.63	0.20
Male	108	96 (14.0%)	12 (20.0%)		
Female	640	592 (86.0%)	48 (80.0%)		
HT				0.42	0.52
No	667	612 (89.0%)	55 (91.7%)		
Yes	81	76 (11.0%)	5 (8.3%)		
Nodular goiters				2.49	0.12
No	397	371 (53.9%)	26 (43.3%)		
Yes	351	317 (46.1%)	34 (56.7%)		
Multifocality				1.61	0.20
No	607	562 (81.7%)	45 (75.0%)		
Yes	141	126 (18.3%)	15 (25.0%)		
Bilaterality				0.00	0.98
No	462	425 (61.8%)	37 (61.7%)		
Yes	286	263 (38.2%)	23 (38.3%)		
Primary tumor size				74.89	< 0.001*
≤ 1 cm	540	520 (75.6%)	20 (33.3%)		
> 1 and ≤ 2 cm	170	145 (21.1%)	25 (41.7%)		
> 2 cm	38	23 (3.3%)	15 (25.0%)		
Capsule contact				31.36	< 0.001^*^
No	275	273 (39.7%)	2 (3.3%)		
Yes	473	415 (60.3%)	58 (96.7%)		
ETE				82.02	< 0.001^*^
No	505	496 (72.1%)	9 (15.0%)		
Yes	243	192 (27.9%)	51 (85.0%)		
SWE imaging					
E_max_	748	54.87 ± 14.55	61.70 ± 11.90	-2.92	< 0.001^*^
E_mean_	748	44.01 ± 11.97	46.80 ± 9.82	-1.36	0.08
ER	748	1.85 ± 0.46	2.16 ± 0.48	-4.97	< 0.001^*^
Central LNM				101.56	< 0.001^*^
No	534	525 (76.3%)	9 (15.0%)		
Yes	214	163 (23.7%)	51 (85.0%)		
Number of pN1				205.02	< 0.001^*^
≤ 5 pN1	707	675 (98.1%)	32 (53.3%)		
> 5 pN1	41	13 (1.9%)	28 (46.7%)		

*HT *Hashimoto's thyroiditis*, ETE* Extrathyroidal extension*, SWE* Shear wave elastography*, E*_*max*_ the maximum elasticity values*, E*_*mean*_ the mean elasticity values*, ER* elastic ratio*, LNM* lymph mode metastasis*, pN1* pathologic N1*,* ^*^ statistically significant

**Table 3:** Risk factors for cN1 in patients with PTC

Variable	Univariate analysis			Multivariate analysis		
	**OR**	**95% CI**	***p***	**OR**	**95% CI**	***p***
Age (reference: ≤ 40	—	—	—			
> 40 and ≤ 55	0.60	0.34 ~ 1.05	0.07			
> 55	0.70	0.28 ~ 1.76	0.44			
Gender (Female vs. Male)	0.65	0.33 ~ 1.27	0.20			
HT (yes vs. no)	0.73	0.28 ~ 1.89	0.52			
Nodular goiters (yes vs. no)	1.53	0.90 ~ 2.61	0.12			
Multifocality (yes vs. no)	1.49	0.80 ~ 2.75	0.21			
Bilaterality (yes vs. no)	1.01	0.58 ~ 1.73	0.98			
Primary tumor size (reference: ≤ 1 cm)	—	—	—			
> 1and ≤ 2 cm	4.48	2.42 ~ 8.30	< 0.001*	1.71	0.80 ~ 3.62	0.17
> 2 cm	16.96	7.70 ~ 37.33	< 0.001*	5.05	1.88 ~ 13.55	0.01*
Capsule contact (yes vs. no)	19.08	4.62 ~ 78.76	< 0.001*	5.77	1.27 ~ 26.30	0.02*
ETE (yes vs. no)	14.64	7.07 ~ 30.32	< 0.001*	4.20	1.78 ~ 9.92	0.01*
E_max_	1.03	1.01 ~ 1.05	0.001*			
E_mean_	1.02	0.99 ~ 1.04	0.08			
ER	3.27	1.99 ~ 5.35	< 0.001*			
Central LNM (yes vs. no)	18.25	8.80 ~ 37.88	< 0.001*	5.18	2.22 ~ 12.06	< 0.001*
Number of pN1 (> 5 vs. ≤ 5)	45.43	21.52 ~ 95.92	< 0.001*	9.52	4.04 ~ 22.43	< 0.001*

*OR* Odds ratio*, CI* Confidence interval

### Association between pN1 and clinicopathological features

**Fig. 1:**
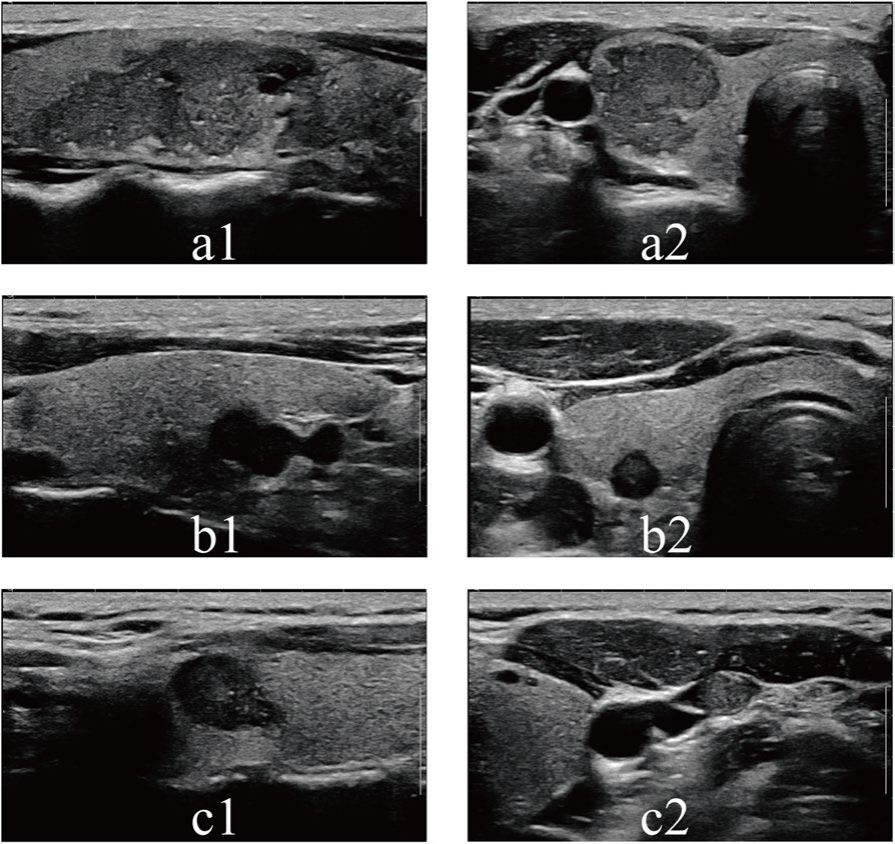
Ultrasound features of the PTC tumor and lymph node: a1 and a2 show tumor size > 2 cm and capsule contact; b1 and b2 show extrathyroidal extensions (ETE); c1 and c2 show capsule contact and lateral metastatic lymph node (III): the lymph node appear enlarged, round

Among the 748 included PTC patients, 707 cases had ≤ 5 LNs involved, and 41 cases had > 5 LNs involved pathologically. Comparisons of the clinicopathological and US characteristics between the 2 groups of patients with PTC are shown in Table 4. Univariate statistical analysis showed that there were significant differences in the multifocality, primary tumor size, capsule contact, ETE, C-TIRADS grade, Emax, ER and central LNM between the two groups (*p* < 0.05). Multivariate logistic regression analysis after univariate statistical analysis demonstrated that multifocality (OR = 2.70 (95% CI 1.31–5.56); *p* = 0.01), primary tumor size > 2 cm (OR = 2.25 (95% CI 1.05–4.83); *p* = 0.01), capsule contact (OR = 4.62 (95% CI 1.70–12.57); *p* = 0.03) and ETE (OR = 4.81 (95% CI 2.09–11.06); *p* < 0.001) were independent risk factors for having > 5 pathological LNs involved (Table 5, Fig. 1).

**Table 4:** Comparisons of clinicopathological features between ≤ 5 pN1 and > 5 pN1 of PTC patients

Variable	Total (*n* = 748)	≤ 5 pN1 (*n* = 707)	> 5 pN1 (*n* = 41)	χ2	*p*
Age(years)				4.24	0.12
≤ 40	246	227 (32.1%)	19 (46.3%)		
> 40 and ≤ 55	423	406 (57.4%)	17 (41.5%)		
> 55	79	74 (10.5%)	5 (12.2%)		
Gender				0.90	0.34
Male	108	100 (14.1%)	8 (19.5%)		
Female	640	607 (85.9%)	33 (80.5%)		
HT				0.00	1.00
No	667	630 (89.1%)	37 (90.2%)		
Yes	81	77 (10.9%)	4 (9.8%)		
Nodular goiters				0.52	0.47
No	397	373 (52.8%)	24 (58.5%)		
Yes	351	334 (47.2%)	17 (41.5%)		
Multifocality				8.92	0.003^*^
No	607	581 (82.2%)	26 (63.4%)		
Yes	141	126 (17.8%)	15 (36.6%)		
Bilaterality				3.10	0.08
No	462	442 (62.5%)	20 (48.8%)		
Yes	286	265 (37.5%)	21 (51.2%)		
Primary tumor size				41.56	< 0.001^*^
≤ 1 cm	540	526 (74.4%)	14 (34.1%)		
> 1 and ≤ 2 cm	170	152 (21.5%)	18 (43.9%)		
> 2 cm	38	29 (4.1%)	9 (22.0%)		
Capsule contact				18.97	< 0.001^*^
No	275	273 (38.6%)	2 (4.9%)		
Yes	473	434 (61.4%)	39 (95.1%)		
ETE				45.57	< 0.001^*^
No	505	497 (70.3%)	8 (19.5%)		
Yes	243	210 (29.7%)	33 (80.5%)		
C-TIRADS				213.6	< 0.001^*^
Category 4a	69	68 (9.5%)	1 (2.4%)		
Category 4b	153	150 (21.2%)	3 (7.3%)		
Category 4c	466	457 (64.6%)	9 (22.0%)		
Category 5	60	32 (4.5%)	28 (68.3%)		
SWE imaging					
E_max_	748	55.05 ± 14.31	61.80 ± 15.89	-2.92	0.004^*^
E_mean_	748	44.09 ± 11.85	46.68 ± 11.38	-1.36	0.17
ER	748	1.86 ± 0.46	2.14 ± 0.54	-3.68	< 0.001^*^
Central LNM				108.24	< 0.001^*^
No	534	534 (75.5%)	0 (0.0%)		
Yes	214	173 (24.5%)	41 (100.0%)

**Table5:** Risk factors for > 5 LN involved in patients with PTC

Variable	Univariate analysis			Multivariate analysis		
	**OR**	**95% CI**	***p***	**OR**	**95% CI**	***p***
Age (reference: ≤ 40)	—	—	—			
> 40 and ≤ 55	0.50	0.26 ~ 0.98	0.04*			
> 55	0.81	0.29 ~ 2.24	0.68			
Gender (Female vs. Male)	0.68	0.31 ~ 1.51	0.34			
HT (yes vs. no)	0.89	0.31 ~ 2.55	0.82			
Nodular goiters (yes vs. no)	0.79	0.42 ~ 1.50	0.47			
Multifocality (yes vs. no)	2.66	1.37 ~ 5.17	0.004*	2.70	1.31 ~ 5.56	0.01*
Bilaterality (yes vs. no)	1.75	0.93 ~ 3.29	0.08			
Primary tumor size (reference: ≤ 1 cm)	—	—	—			
> 1 and ≤ 2 cm	4.45	2.16 ~ 9.15	< 0.001*	2.25	1.05 ~ 4.83	0.05
> 2 cm	11.66	4.66 ~ 29.17	< 0.001*	4.62	1.70 ~ 12.57	0.01*
Capsule contact (yes vs. no)	12.27	2.94 ~ 51.21	0.001*	5.08	1.16 ~ 22.24	0.03*
ETE (yes vs. no)	9.76	4.44 ~ 21.49	< 0.001*	4.80	2.09 ~ 11.06	< 0.001*
E_max_	1.03	1.01 ~ 1.05	0.004*			
E_mean_	1.02	0.99 ~ 1.05	0.17			
ER	2.85	1.60 ~ 5.088	< 0.001*

## Discussion

Although the metastatic spread of PTC to the cervical LNs does not seem to affect overall or disease-specific survival, it does increase the risk of local or regional recurrence of the tumor and the need for further surgery and follow-up. The evaluation of cervical LNM is very important for determining the appropriate extent of LND and patient prognosis. Because all LNM do not affect the prognosis of patients equally [8], the need for the risk stratification of LNM has been suggested. The risk of structural disease recurrence varies according to the presence of clinically apparent LNs, the number of LN metastases, and the presence of ENE [9, 10]. Now the 2015 ATA guidelines are the most commonly used for PTC, its ability to predict persistent disease and recurrence has been validated through three tiers risk stratification system. The 2015 ATA guidelines classified cN1 and > 5 pN1 as having an intermediate risk. In patients with intermediate risk PTC, the risk of persistent disease and recurrence was higher than in patients with low-risk PTC (20% vs. 5%). The parameters of cN1 and > 5 pN1 were not included in the original 2009 initial risk stratification system. A few studies have attempted to explore the association of LNM with clinicopathologic and ultrasound features of the primary tumor [11, 12]. Thus, it would be useful to examine preoperative cN1 and pN1 data to predict intermediate risk in PTC patients. The results showed that multifocality, primary tumor size > 2 cm, capsule contact, ETE and central LNM were independent risk factors for intermediate risk in PTC patients.

This study shows that primary tumor size > 2 cm, capsule contact, ETE and central LNM independent affected cN1. Cervical LNMs are quite common in the cN0 patients, they usually are very small in size and number. They can't be detected before surgery. cN1 were identified by preoperative physical examination, US, CT imaging and/or intraoperative inspection. In the clinic, thyroid disease and LNs are commonly detected during preoperative evaluation by US. US can directly and accurately detect lateral cervical LNM and obvious central LNM. If central metastatic nodes are masked by surrounding structures in the central neck, clavicle and sternum, US can determine the status of the thyroid nodule. Although US results cannot replace pathological results, most studies have found that ultrasound results are consistent with pathological results [13]. When preoperative US show primary tumor size > 2 cm and capsule contact, they may indicate cN1 and an intermediate risk.

The study shows that multifocality, primary tumor size > 2 cm, capsule contact and ETE are risk factors the pathological involvement of > 5 LNs. Fortunately, some risks of LNM can be detected preoperatively with US, such as multifocality, primary tumor size, and capsule contact and gross ETE. However, other risks, such as minor ETE, are often only identified in the final surgical pathology. Several researchers have similarly found the prognostic importance of ETE. ETE is a strong predictor of clinical outcome and is an important prognostic factor in PTC patients [14]. Ito et al. showed that ETE was associated with worse disease-free survival and cause-specific survival [15, 16]. This prognostic effect of ETE, which is greater than that of multifocality, primary tumor size and capsule contact, has implications for future updates of the PTC classification of the TNM system from stage T1 or T2 to stage T3. According to research, the ETE of tumors is known to be associated with cancer cell proliferation and invasiveness, and greater invasiveness is usually correlated with a higher risk of LNM. For multifocality, in the present study, the incidence of multifocality was comparable to previously reported rates, ranging from 6.7% to 14.6%, and this factor was identified as the sole predictor of recurrence [17]. This was partially because more malignant neoplasms increase the chance of lymphatic spread. Preoperative multifocality US evaluation was highly consistent with postoperative pathology. Studies have shown that multifocality is associated with central LNM and not with lateral LNM [18]. Because central LNs may not be easily found clinically due to being masked by a surrounding structure, this may be why multifocality is associated with pN1 and not with cN1.

Characteristics of the LNM can further discriminate the risk of recurrence, especially in intermediate risk patients with clinically evident metastasis and multiple metastases [5]. The aim of the study was to identify clinicopathologic and US features of PTC for intermediate risk of cN1 and pN5. We concluded that multifocality, primary tumor size > 2 cm, capsule contact, ETE and central LNM were independent risk factors for intermediate risk. Once identified as having an intermediate-risk, particularly through preoperative US, the treatment may begin immediately and can be aggressive, involving total thyroidectomy and prophylactic LND and RAI administration or active follow-up strategies. If identified as having a low-risk, active surveillance, ablation treatment or thyroid lobectomy could be taken. Clinical and ultrasound factors that revealed preoperatively is a crucial element in the management of the PTC patients. Also, this finding can help the surgeon plan the extent of surgery, resulting in a reduction in the number of total thyroidectomy, which is associated with potentially greater complications than lobectomy.

The prognosis was not analyzed. There may be local or regional LNs recurrence in some cases during follow-up. Many studies were the conclusions that the effect of the presence of LNM on overall survival is small. The relatively good overall prognosis of the ATA intermediate risk group are important considerations in RAI decision-making. We advise that the strategy for treating patients with intermediate-risk PTC should be defined on a case-by-case basis.

This study had some limitations. First, the most important limitation was the retrospective design of the study, which may have led to selection bias and the loss of some clinicopathological characteristics. Second, the prediction of recurrence in patients with intermediate-risk PTC should be further developed. The data should give a thought to the location and size of metastatic foci. Third, we did not classify the PTC patients either by basing on histological subtypes or dividing LN metastases (N1a and N1b), which may be associated with intermediate risk of recurrence. At last, the number of patients included represents a relatively small cohort in a single center. To ensure a larger and more representative sample, multicenter studies are required.

## Conclusions

In the study, the incorporation of the 2015 ATA guidelines into our practice has obtained patients with intermediate-risk PTC to stratified risk factors of clinicopathological features. We concluded that multifocality, primary tumor size > 2 cm, capsule contact, ETE and central LNM were independent risk factors for both cN1 and > 5 pN1 and the risk factors were related to intermediate risk. Clinical and US factors including multifocality, primary tumor size > 2 cm, capsule contact and gross ETE that revealed preoperatively are crucial in PTC patients. Now, a major unresolved problem is how to manage patients with intermediate-risk PTC. At least, ultrasonography is a good technique for the preoperative LN staging of PTC and is helpful for detecting LNM in the central and lateral group.

## Data Availability

The datasets used and/or analyzed during the current study are available from the corresponding author on reasonable request.

## Abbreviations

PTC: Papillary thyroid carcinoma
LN: Lymph node
LNM: Lymph mode metastasis
AJCC8th: 8Th edition of the American Joint Commission on Cancer
DTC: Differentiated thyroid cancer
ATA: American Thyroid Association
cN1: Clinical N1
pN1: Pathologic N1
LND: Lymph node dissection
HT: Hashimoto's thyroiditis
ETE: Extrathyroidal extensions
SWE: Shear wave elastography
C-TIRADS: Chinese Thyroid Imaging Reporting and Data System
FNA: Fine needle aspiration
RAI: Postsurgical radioiodine
CT: Computed tomography
cN0: Clinical N0
ENE: Extranodal extension
TNM: Tumor node metastasis
